# The Rehabilitation Program Improves Balance Control in Children with Excessive Body Weight and Flat Feet by Activating the Intrinsic Muscles of the Foot: A Preliminary Study

**DOI:** 10.3390/jcm12103364

**Published:** 2023-05-09

**Authors:** Maria Markowicz, Wojciech Skrobot, Agnieszka Łabuć, Paulina Poszytek, Agnieszka Orlikowska, Ewelina Perzanowska, Katarzyna Krasowska, Konrad Drewek, Jan J. Kaczor

**Affiliations:** 1 Department of Health and Life Sciences, Department of Clinical Physiotherapy, Faculty of Physical Education, Gdansk University of Physical Education and Sport, 80-336 Gdansk, Poland; 2 Cathedral and Clinic for Orthopaedics and Traumatology, Medical University of Gdansk, 80-210 Gdansk, Poland; 3 Division of Bioenergetics and Physiology of Exercise, Medical University of Gdansk, 80-210 Gdansk, Poland

**Keywords:** flat foot, excessive body weight, balance control, intrinsic foot muscles, arch height, medial longitudinal arch

## Abstract

Background: determining the appropriate rehabilitation protocol is essential to influence the correction of flat feet, e.g., by activating the intrinsic muscles of the foot. Therefore, this study aimed to determine the impact of the exercises activating the intrinsic foot muscles for postural control in children with flat feet, with normal and excessive body weight. Methods: Fifty-four children aged 7 to 12 were enrolled in the research. Forty-five children were qualified for the final evaluation. Each child in the experimental group was demonstrated an appropriate technique for performing a short foot exercise without compensation by extrinsic muscle. The participants then performed a supervised short foot training session once a week and on other days of the week under the supervision of caregivers for 6 weeks. Flat feet were scored on the foot posture index scale. A postural test was evaluated with a Biodex balance system SD. Statistical significance in the foot posture index scale and postural test were evaluated using an analysis of variance (ANOVA) with Tukey’s post-hoc test. Results: according to the six indices of the foot posture index scale, five indicators showed statistically significant improvement after rehabilitation. At the 8–12 platform mobility level, it was revealed that the excessive body weight group had significant improvements in the overall stability index and medio-lateral stability index, with eyes closed. Conclusion: our results indicate that a 6-week rehabilitation program based on the activation of the intrinsic muscles of the foot resulted in an improvement in the foot position. This, in turn, affected balance control, especially in children with excess body weight in conditions of closed eyes.

## 1. Introduction

In pediatric patients, flat feet are one of the predominant problems and one of the most common reasons for visiting a specialist [1], the problem mainly concerns the medial longitudinal arch (MLA) [2]. As a medical condition, flat foot is defined by the absence of a reduced medial longitudinal arch, with osseo-ligamentous misalignment. While weight-bearing, the heel is in the valgus, flattening the medial longitudinal arch and causing deviation of the forefoot in abduction [3]. As the most distal segment of the lower limb bio-kinematic chain, the foot represents a relatively small support base while maintaining balance. Even the slightest changes in this segment may cause disturbances in the posture control strategy [4]. Flat feet affect the entire kinematic chain and cause functional instability of the foot, combined with impaired balance disorders, issues with proprioception, and changes in the proximal joints [5]. An important factor that can cause or worsen this disadvantage is obesity. This is because the lower limb is subjected to additional weight, and the foot must support approximately 0.5 times more body weight when standing [6]. This increased burden on the MLA may lead to an imbalance [7]. Therefore, obese children are more likely to develop flat foot [8]. Hitherto, research has focused on the links between obesity and flat feet and postural stability control [7,9,10,11,12]. Therefore, it is necessary to emphasize the need to create an appropriate rehabilitation protocol that would reduce the effects of flat feet.

Regarding the functioning of various flat feet exercise programs, they are considered to emphasize the importance of the intrinsic muscles of the foot and their impact on improving the function of flat feet [5,13,14,15,16,17]. The plantar intrinsic foot muscles are the most important muscles to maintain the arch of the foot [18,19,20,21,22]. The intrinsic foot muscles may contribute to the absorption of power in the early stance and the recovery and generation of power in the late stance [23]. The approach to treating flat feet has changed over the years and many solutions remain, including treatment with orthotics, insoles, kinesiotaping, exercise protocols focusing on extrinsic foot muscles or internal foot muscles, and the use of the PNF concept [13,24,25,26]. It should also be emphasized that daily physical activity in children affects the process of foot arch development through the desired bone development and mineralization, as well as an increase in flexibility and muscle strength. There is a strong relationship between the endurance and strength of the muscles of the lower limbs and the development of the arches of the foot. Truszczyńska-Baszak and coauthors showed that the percentage of normal and high foot arches increased with increasing levels of physical activity and physical fitness. In turn, a lowered longitudinal foot arch might reduce physical activity and fitness in adolescents [27]. Therefore, the advantage of using an appropriate exercise program, which is also part of this rehabilitation program, should be emphasized.

The results of electromyographic studies in flat foot conditions indicate compensatory, increased activity of the external muscles of the foot, and decreased activity of the intrinsic foot muscles [20,21,22]. Therefore, this study focused on training the intrinsic muscles of the foot. ‘Short foot exercises’ provide isolated tension of intrinsic foot muscles; they are frequently overlooked in therapy. Training the intrinsic muscles of the foot may improve the function of the foot [17,19]. McKeon and coworkers showed disturbances in the activity of the muscles responsible for the stabilization of the arches of the foot [19]. Moreover, other scientists have noticed similar observations [28,29]. Preadolescent age seems to be an important time to evaluate the foot structure and manage residual abnormalities not resolved by the developmental processes before 6 years, as after puberty the possibility of correction of the medial longitudinal arch of the foot will be significantly reduced [30]. Children who do not develop MLA adequately by age 10 may have limited potential for flat feet to resolve naturally [31].

The process of rehabilitation of children with excessive body weight and flat feet has not been sufficiently explored, despite the frequent occurrence of flat feet and obesity in children.

Moreover, to our knowledge, there is not enough evidence showing the effectiveness of the rehabilitation of flat feet in children with excess body weight in the literature. Therefore, this study aimed to determine the impact of the 6-week rehabilitation program, consisting of exercises activating the intrinsic foot muscles for postural control in children with flat feet, with normal and excessive body mass.

## 2. Materials and Methods

The study was conducted in accordance with the Declaration of Helsinki, and approved by the Independent Bioethics Committee for Scientific Research at the Medical University of Gdansk. Resolution number NKBBN/716/2019-2020. The trial was registered in Clinical Trials and given the number NCT04840017. Parents or legal guardians of all participants gave their written informed consent to participate in this study.

This publication complies with the Transparent Reporting of Evaluations with Nonrandomized Designs (TREND) checklist. The study was not randomized.

### 2.1. Subjects

**Figure 1:**
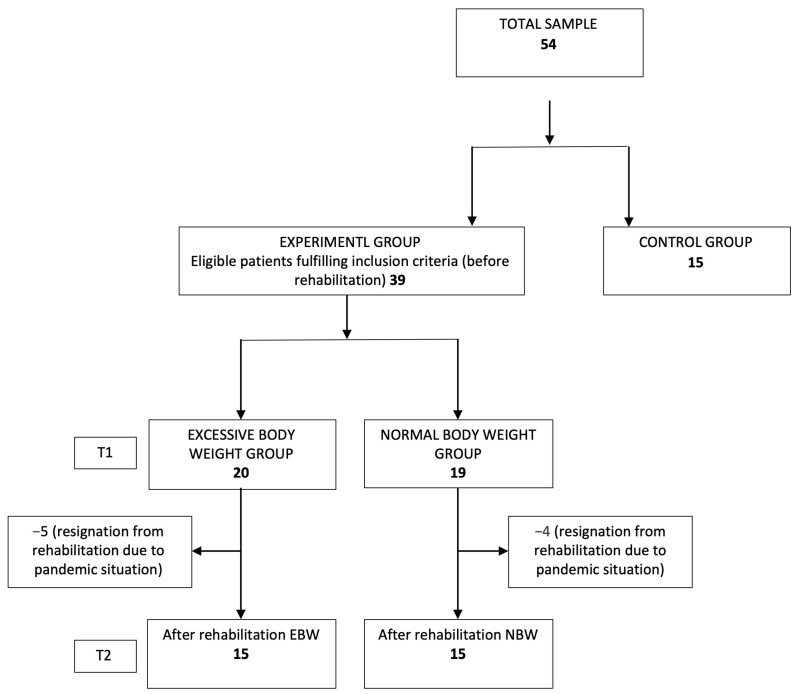
Participants flow diagram. T1—condition before rehabilitation, T2—condition after rehabilitation. In the EBW group, three patients did not undergo the rehabilitation process, two resigned due to the pandemic situation. In the NBW group, four patients did not complete the rehabilitation process due to the pandemic situation.

Thirty-nine (39) children aged 7 to 12 were enrolled in the experimental groups. The age of the children was selected based on the literature [30]. The children came from urban areas. The children were divided into two groups: the normal body weight group (NBW), and the excessive body weight group (EBW), and 15 children were recruited to the control group (CG) (Figure 1).

#### 2.1.1. Characteristics of the Groups

In the NBW and EBW groups, there were children with bilateral, symptomatic flexible flatfeet. The CG consisted of healthy children. The NBW group consisted of 10 boys and 5 girls, and 9 boys and 6 girls constituted the EBW group. The CG consisted of 8 boys and 7 girls. The girls in the study have not yet started the menstrual cycle. For this reason, the influence of hormones on the test results was not considered.

#### 2.1.2. Inclusion Criteria

Children with bilateral, symptomatic flexible flat feet were included in the study.

#### 2.1.3. Exclusion Criteria

Children with tarsal coalitions, congenital defects of lower limbs, neurological diseases, and previous foot surgery were excluded from the study.

### 2.2. Examination

Each child was examined by an orthopedist who classified them for symptomatic flexible flat feet. The children also underwent a functional physiotherapeutic examination. The weight and height of each child were assessed to determine body mass index (BMI) according to international cut-off points [32], and body composition was further analyzed by bioelectric impedance. Each research procedure was performed twice: before rehabilitation (T1, EBW1, NBW1) and after a 6-week rehabilitation program (T2, EBW2, NBW2). The examination was performed each time by the same, qualified, and independent physiotherapist. This physiotherapist did not participate in the rehabilitation process. Data collection and intervention were carried out at the University of Physical Education and Sport, Gdansk.

### 2.3. Tests

#### 2.3.1. Foot Posture Index Scale (FPI-6)

FPI-6 is a clinical tool for quantifying foot positioning that uses established criteria. During the evaluation each child stands with their bare feet slightly apart, with both hands at the sides of the body, looking straight ahead. Assessment consists of 6 separate scoring sections that are summarized to provide a score that reflects the position of the foot in space. Each of the 6 parts is graded on a scale of −2 to 2.

The evaluation was performed in the following order:

- Talar Head Palpation
- Supra and infra lateral malleoli curvature (viewed from behind)
- Calcaneal frontal plane position (viewed from behind)
- Prominence in the region of the talonavicular joint (TNJ) (viewed at an angle from the inside)
- Congruence of the medial longitudinal arch (viewed from inside)
- Abduction/adduction of the forefoot on rearfoot (viewed from behind)

The sum of points designated the neutral position of the foot should be in the range from 0 to 5, from 6 to 12 points indicate pronation of the foot and the range from −1 to −12 indicates a supination position [33].

#### 2.3.2. Postural Stability Test

The postural stability test was evaluated with a Biodex balance system SD 115VAC [34]. Postural stability control was performed on an unstable platform using stiffness levels 5 and 12 to 8 levels (12 as the most stable platform, 1 as the least). The platform stability range 5 and 8–12 is considered a fall risk test. Each test consisted of three attempts: 20 s of each sample, and 10 s of break. Familiarization was performed before the test, and after explaining the test procedure. The patient’s age, height, and the position of feet with the third metatarsal bone as well as the position of the heels were entered into the platform. The trial was invalid if the patient removes the foot from the platform. Three indicators were obtained: Anterior-Posterior Stability Index (APSI), Medio-Lateral Stability Index (MLSI), and Overall Stability Index (OSI).

During the evaluation each child was asked to stand in the center of the platform with their bare feet with both hands at the sides of the body, looking straight ahead, focusing on the visual feedback screen. For each level of measured dynamic stability, three trials were performed from which the average was calculated. The children were examined in two states: with open and closed eyes.

### 2.4. Intervention

**Figure 2:**
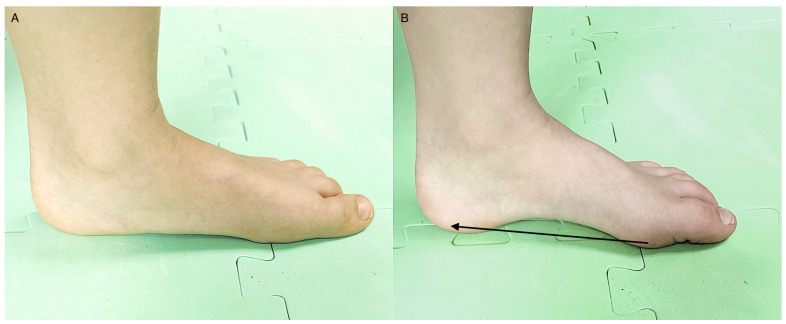
Short foot exercise. (**A**) flat foot without activation of intrinsic muscles, (**B**) the correct technique consisting in performing the a “foot shortening” and MLA elevation by drawing the first metatarsal head towards the calcaneus.

Each participant from the experimental group demonstrated an appropriate short foot exercise (SFE) technique [35] without compensation by the extrinsic muscle (Figure 2). Each subject performed a supervised short foot training session once a week, and an unsupervised SFE each day for 6 weeks. Details of the rehabilitation protocol are presented in Table 1.

The rehabilitation program was always supervised by the same, qualified, and independent physiotherapist. During supervised rehabilitation, participants were taught SFE techniques and the subsequent exercise progressions. Exercise progression includes, among others, changes in the initial positions. Each exercise consisted of twenty repetitions. Each repetition was held for twenty seconds, and there was also a twenty-second rest between repetitions. Supervised rehabilitation was performed with the participation of parents, who were familiar with the correct methodology for each exercise as well as the children, and knew the number of repetitions, series, tension time, and break time between exercises. Parents were also asked to pay attention to any compensatory movements or abnormalities during each task and to encourage exercise. Therefore, they were able to supervise their children’s exercise at home. The session was conducted until each participant was able to correctly complete each exercise. When it was noticed that the exercise was not performed correctly (e.g., due to fatigue), a break was taken, and then the learning was repeated. The length of the meeting was adjusted to the individual needs of each individual.

Subjects were asked to maintain their regular levels of activity and not introduce any new training routines during the study. None of the children attended additional sports activities or additional rehabilitation during the study period. The children in the study never participated in rehabilitation due to flat feet. There were also no children from sports schools or children attending sports activities more than once a week. A questionnaire on the actual amount of home exercise program performance was drafted and the results are presented in Table 2.

### 2.5. Statistical Analysis

Statistical analyses were performed using a software package Statistical 13.3, StatSoft Inc., Tulsa, OK, USA. Statistical significance in FPI-6 and Postural Stability Test was evaluated using an analysis of variance (ANOVA) with Tukey’s post-hoc test. The *t*-test was used to compare the demographic properties of the examined children. *p* < 0.05 was considered statistically significant. Moreover, the Pearson correlation between BMI and the Postural Stability Test was checked.

Using G*Power 3.1 software, Heinrich Heine Universität Düsseldorf: Psychologie, Germany for performing a sample size calculation, we estimated a priori that we needed at least 22 participants (11 per group) to detect significant differences in FPI-6 and the Postural Stability Test. This sample size was estimated using *p* values = 0.05, power = 0.8, effect size = 0.25, and number of groups = 2, number of measurements = 2.

## 3. Results

### 3.1. The Demographic Data of Children

The demographic data of the children in this study are summarized in Table 3. There were no statistically significant differences between the EBW, NBW, and CG groups in terms of age. Regarding BMI, there were no statistically significant differences between the CG and the NBW group. The EBW group showed a significantly higher BMI than the other groups. Moreover, during the 6-week rehabilitation, no statistically significant changes in children’s BMI were observed.

### 3.2. Foot Posture Index Scale (FPI-6)

According to the six indicators of the FPI-6, five indicators presented statistically significant differences. Before the intervention, the values obtained in the NBW and EBW groups indicated a pronated foot posture and collapse of the medial longitudinal arch.

The rehabilitation program improved the position of the foot on palpation of the head of the talus, supra, and infra lateral malleoli curvature, calcaneal frontal plane position, and congruence of the medial longitudinal arch in both EBW and NBW groups compared to the initial measures.

**Figure 3:**
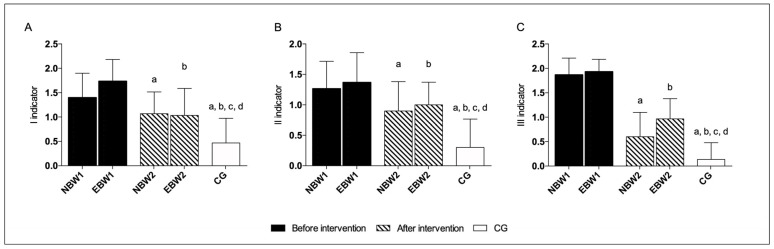
FPI-6 indicators I-III. The data are presented as the means with standard deviations (SDs). a, significantly different compared with NBW1; b, significantly different with EBW1; c, significantly different compared with NBW2; d, significantly different with EBW2. (**A**) talar head palpation (a, NBW2 *p* = 0.001; a, CG *p* = 0.0002; b, EBW2 *p* = 0.0001; b, CG *p* = 0.00002; c, CG *p* = 0.00006; d, CG *p* = 0.0002); (**B**) supra and infra lateral malleoli curvature (a, NBW2 *p* = 0.0001; a, CG *p* = 0.00002; b, EBW2 *p* = 0.0001; b, CG *p* = 0.00002; c, CG *p* = 0.00002; d, CG *p* = 0.00002); (**C**) calcaneal frontal plane position (a, NBW2 *p* = 0.0001; a, CG *p* = 0.00002; b, EBW2 *p* = 0.0001; b, CG *p* = 0.00002; c, CG *p* = 0.00003; d, CG *p* = 0.00002). The NBW1, EBW1 groups before rehabilitation, and the NBW2, EBW2 groups after rehabilitation, and CG.

**Figure 4:**
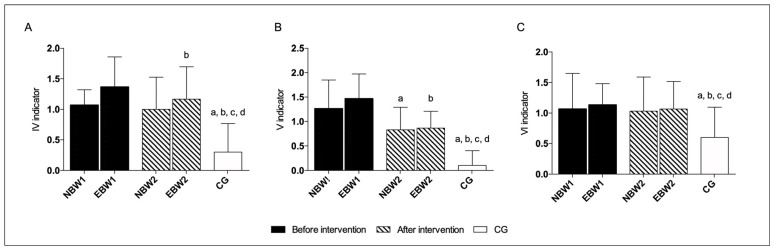
FPI-6 indicators IV-VI. The data are presented as the means with standard deviations (SDs). a, significantly different compared with NBW1; b, significantly different with EBW1; c, significantly different compared with NBW2; d, significantly different with EBW2. (**A**) prominence in the region of the TNJ (a, CG *p* = 0.0001; b, EBW2 *p* = 0.028; b, CG *p* = 0.0001; c, CG *p* = 0.0001; d, CG *p* = 0.0001); (**B**) congruence of the MLA (a, NBW2 *p* = 0.0001; a, CG *p* = 0.00002; b, EBW2 *p* = 0.0001; b, CG *p* = 0.00002; c, CG *p* = 0.00002; d, CG *p* = 0.00002); and (**C**) abduction/adduction of the forefoot on rearfoot (a, CG *p* = 0.005; b, CG *p* = 0.0009; c, CG *p* = 0.01; d, CG *p* = 0.005). The NBW1, EBW1 groups before rehabilitation and the NBW2, EBW2 groups after rehabilitation, and CG.

**Figure 5:**
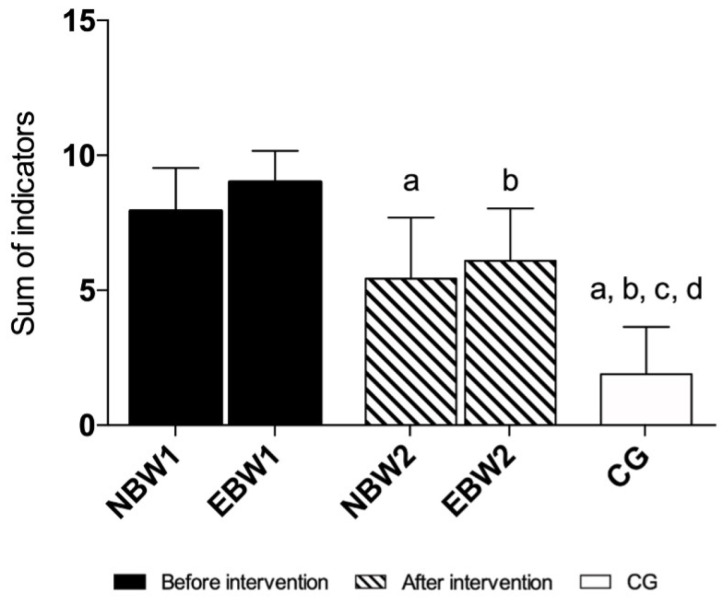
The sum of FPI-6 indicators. The data are presented as the means with standard deviations (SDs). a, *p* = 0.0001, significantly different compared with NBW1; b, significantly different with EBW1; c, significantly different compared with NBW2; d, significantly different with EBW2. (a, NBW2 *p* = 0.0001; a, CG *p* = 0.0001; b, EBW2 *p* = 0.0001; b, CG *p* = 0.0001; c, CG *p* = 0.0001; d, CG *p* = 0.0001).

Additionally, in the EBW group, the prominence in the region of the talonavicular joint was close to 0, which indicated a more neutral foot position. The CG values indicated a neutral position of the foot and the correct shape of the medial longitudinal arch (Figure 3, Figure 4 and Figure 5).

### 3.3. Postural Stability Test

A stability index in the range of 8–12 was compared in the risk of fall test, before and after the rehabilitation program.

**Figure 6:**
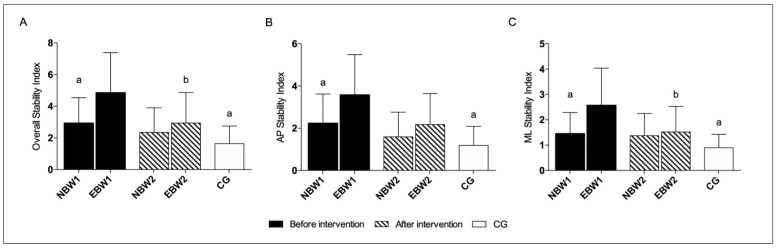
The stability indexes with eyes closed range 8–12. The data are presented as the means with SDs. a, significantly different with EBW1; b, significantly different with EBW1. (**A**) Overall Stability Index (a, NBW1 *p* = 0.012; a, CG *p* = 0.0002; b, EBW2 *p* = 0.031); (**B**) Anterior-Posterior Stability Index (a, NBW1 *p* = 0.025; a, CG *p* = 0.0003); (**C**) Medio-Lateral Stability Index (a, NBW1 *p* = 0.014; a, CG *p* = 0.0004; b, EBW2 *p* = 0.028).

It was revealed that the EBW group had significant improvements after rehabilitation in the OSI and MLSI, with eyes closed (Figure 6). In the NBW group, most indicators showed a tendency to improve in this area of mobility, with eyes open and closed, but without statistically significant differences (Table 4).

In all indexes: OSI, APSI, and MLSI with eyes closed before rehabilitation, the EBW group differed statistically significantly to the NBW group.

The measurements in the EBW group showed significant differences in all indexes as compared to the above results with the CG before rehabilitation. After the intervention, for the range of platform mobility 8–12, in APSI and MLSI indexes, with eyes closed, the EBW group reached similar reference values.

There were no significant differences between the CG and NBW group, neither before nor after the rehabilitation program, with eyes closed, at this range of mobility (Figure 6).

**Figure 7:**
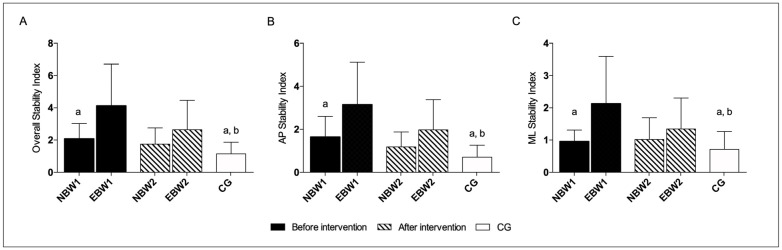
The stability indexes with eyes closed range 5. The data are presented as the means with SDs. a, significantly different with EBW1, b, significantly different with EBW2. (**A**) Overall Stability Index (a, NBW1 *p* = 0.002; a, CG *p* = 0.0002; b, CG *p* = 0.023); (**B**) Anterior-Posterior Stability Index (a, NBW1 *p* = 0.006; a, CG *p* = 0.0001; b, CG *p* = 0.037); (**C**) Medio-Lateral Stability Index (a, NBW1 *p* = 0.005; a, CG *p* = 0.001; b, CG *p* = 0.032).

At the fifth platform mobility level with eyes open and closed, no statistically significant differences were found in the NBW and EBW group as compared with the period before to after the intervention (Figure 7 and Table 5).

The OSI, APSI, and MLSI in the EBW group at the fifth platform mobility level with eyes closed appeared to show statistically significant differences, before rehabilitation, compared to the NBW group and CG. After rehabilitation, the EBW group approached the values achieved by the NBW group but did not reach the reference values understood as CG. There were no significant differences between the CG and NBW groups both, after and before the intervention (Figure 7).

### 3.4. Pearson Linear Correlation

**Figure 8:**
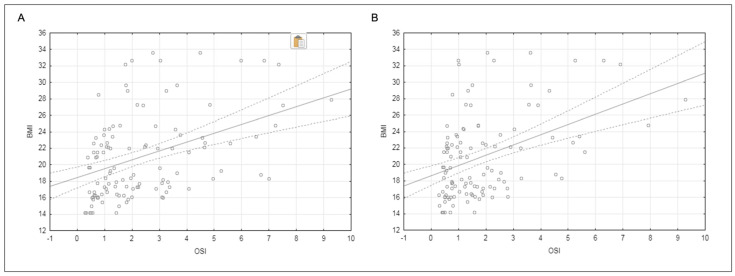
Pearson linear correlation between BMI and OSI (**A**) in tests 8–12. r = 0.43, significantly different *p* = 0.0001, (**B**) in fifth platform mobility level. r = 0.44, significantly different *p* = 0.0001.

The general correlation between BMI and OSI in tests 8–12 was positive and statistically significant (r = 0.43, *p* = 0.0001). In addition, the correlation between BMI and OSI at the fifth platform mobility level was also positive and statistically significant (r = 0.44, *p* = 0.0001) (Figure 8). Whereas the correlation of BMI and OSI eyes closed in both tests 8–12 and 5 was even stronger, respectively, r = 0.55, *p* = 0.0001, and r = 0.599, *p* = 0.0001. Additionally, the correlation of BMI and OSI eyes open in both tests 8–12 and 5 was weaker, respectively, r = 0.42, *p* = 0.001, and r = 0.35, *p* = 0.006 (Table 6).

## 4. Discussion

The main aim of this study was to investigate the impact of the application of exercise activating the intrinsic foot muscles in children with flat feet and their effect on the shape of the foot, mainly changes in the MLA and balance control. We found that the 6-week rehabilitation program had a positive effect on the correction of the foot position and control of balance in children with excessive as well as normal body weight. Moreover, our results also showed that exercise activating the intrinsic foot muscles improved the status and shape of the foot in children’s feet.

In flat pronated feet, lowering the arches among others tightens the plantar ligaments and the plantar fascia. Moreover, prolonged pressure on these structures may cause a cycle of microtears, pain, and inflammation [36,37]. Furthermore, flat feet may affect proprioception and sense of balance. Cote and coworkers showed that excessive pronation may affect peripheral, afferent somatosensory signals through changes in joint mobility or contact surface or by changing muscle strategies to maintain a stable base of support [4]. In a meta-analysis of randomized controlled trials to examine the effects of the SFE compared to foot orthosis or other types of interventions, it was found that the SFE may contribute more benefits than other interventions as it affects flat foot individuals’ foot alignment. Hence, the SFE is recommended as a beneficial dynamic support when facing flat foot problems [38]. Therefore, a specific form of rehabilitation is necessary for the control and improvement of the MLA and foot pronation. Among the different concepts of rehabilitation programs [24,39,40,41,42], the dominant goal is to directly influence the shape of the foot arch—the short intrinsic muscles of the foot. Kelly et al. demonstrated that electrical stimulation of the intrinsic foot muscles counteracts the deformation that occurred due to the application of an external load, by shortening the length and increasing the height of the MLA. These findings show that these muscles can control foot position and MLA stiffness, and can improve the effect of support when the foot is loaded. This active arch stiffening mechanism may have an important role in the way forces transmission during locomotor and postural activities [43].

When assessing the FPI-6 indicators, and compared the condition before rehabilitation (T1) to the condition after rehabilitation (T2), significant differences are noticeable in five in the EBW group and four indicators in the NBW group. Moreover, the sum of the FPI-6 scale in both groups showed meaningful differences. The groups after rehabilitation approached the reference values obtained by the control group, which was characterized by the correct positioning of the foot. Generally, our study shows that the improvement in the activity of the intrinsic muscles of the foot was confirmed by a significant change in the foot position and shape of the MLA, expressed with the FPI-6 scale. Our study is in line with the work of Okamura and coauthors, where it was reported that an 8-week training focused on the intrinsic muscles of the foot improved the sum of FPI-6 indicators [44]. The obtained results in the current study also agree with a randomized study by Brijwasi and colleagues where, after 6 weeks of a comprehensive exercise program including an amount of other short foot exercises, the flexible flat feet experimental group improved the navicular height and longitudinal arch angle more than active dorsiflexion and plantar flexion in the control group [45].

In confirmation with our and other research [5], flat feet are related to a balance disorder. In another study, Wright and coauthors emphasized that the foot is an important receptive field. Many cutaneous and load-related proprioceptive receptors may be involved in the appropriate control of foot position or alignment during walking. The foot as the base of support is considered the body’s reference point in space, similarly to how external objects can be incorporated into our body schema. The authors found that the influence of slope changes in the metatarsal and toe areas affects the postural sway, suggesting that the foot, instead of serving as a rigid base of support, is in an active, flexible state and is sensitive to minor disturbances [46]. Tahmasebi and coworkers found a significant difference between the stability of flat-arched and normal individuals based on the center of pressure velocity [47]. An additional factor influencing postural control in children with flat feet is the frequent excess body weight. Being overweight in childhood causes reduced motor functionality and problems with posture control [48]. Deforche et al. proved that obese boys presented a decreased capability of performing tasks requiring static and dynamic balance [12]. Moreover, Hue et al. reported a negative correlation between postural stability and excess body weight [7]. Hence, the present study clearly shows differences in the comparison of the NBW and EBW groups and concerning CG. A very interesting observation is the change in the stability coefficient after rehabilitation for different rigidity settings of the platform. In the EBW group with eyes closed, we observed a significant improvement, especially in terms of the mobility of platforms 8–12, concerning OSI and MLSI. There was a noticeable improvement trend in the NBW group. Similar observations concern the postural stability index of the setting of the unstable platform level 5, we noticed a clear but not significant tendency to improve compared to the CG.

Our results are in agreement with previous studies. Mashhadi et al. researched the effectiveness of the foot shortening maneuver in children and the impact of this maneuver on the behavior of MLA. Exercises are recommended to improve the parameters of the foot arch [17]. Zhang et al. showed that the morphology of the foot muscles plays an important role in maintaining balance and that strengthening the intrinsic foot muscles may be an effective way to improve balance [49]. In another study, Mulligan and coworkers presented the effect of a four-week training of the intrinsic muscles of the foot, in adults, the results were assessed by measuring the height of the tuberosity of the navicular bone and the arch height index. This study indicated an improvement in the balance [50]. It should be noted that most of the studies mentioned were conducted on adults.

Our results indicate an increased involvement of the proprioceptive system, due to significant differences, especially with eyes closed, in the EBW group. The improvement of MLA and the reduction of foot pronation influenced the advancement of the entire biokinematic chain, the effects of which are noticeable in the improvement of balance control and proprioception. As reported by Wright and coworkers, all the neurophysiology and anatomical architecture of the foot is uniquely designed to integrally perform the complex task of bipedal postural control [46]. There are several possible explanations for this involvement of systems. It is noted by the authors that in obesity and flat feet conditions, the contribution of foot mechanoreceptors and skin sensation to balance control changes. Higher pressures and larger foot contact surfaces may reduce the quality and/or quantity of sensory information from plantar mechanoreceptors [7,51,52]. Moreover, the subtalar and ankle joints are surrounded by many protectors having a large proprioceptive role. This is highlighted by research into the role of proprioception after an ankle sprain [53]. In addition, research works on connective tissue show that, for example, the retinaculum is not the structure that statically stabilizes the joints, but they are the specialized structure for local spatial proprioception of foot and ankle movements [54]. Changing the position of the foot may alter the signals received by these receptors.

After rehabilitation, a considerable improvement in foot position and an increase in MLA were noticed. This may indicate a better ability to stabilize the subtalar and ankle joints and the work of receptors located in this area, hence the expected statistically significant improvement in balance control [7,13,14].

In the current study, we found a significant positive overall correlation between BMI and OSI. This shows that the greater the body weight, the worse the balance control. Therefore, our results are consistent with the previous report [7]. However, some separate observations were made by Błaszczyk et al., where obesity influenced to a different extent the characteristics of the center of foot pressure swinging and the anterior limits of stability. In these individuals, weight gain resulted in functional adaptation to control upright posture. It was characterized by reduced swaying posture. This suggests that their postural balance may be well preserved [55].

To the best of the author’s knowledge, this is the first study that assessed the impact of the activation of the intrinsic foot muscles on the correction of flat feet and balance control in children with excess body weight. The conscious control of the foot position and balance has improved as a result of the use of an appropriate exercise program.

One of the limitations of the study was the limited number of participants caused by the COVID-19 pandemic. Secondly, another limitation was the lack of the possibility of randomization. Another one was the lack of children’s diet information. In addition, due to limitations mainly related to the pandemic, it was not possible to perform a follow-up.

## 5. Conclusions

Our results indicate that a 6-week rehabilitation program based on the activation of the intrinsic muscles of the foot resulted in an improvement in the foot position and an increase in MLA in both the EBW group and the NBW group. This, in turn, affected balance control, especially in children with excess body weight in conditions of closed eyes. In the longer term, research will focus on further comparing the group with excessive and normal body weight in a larger population, taking into account randomization and covering a longer follow-up period.

## Figures and Tables

**Table 1:** The details of the rehabilitation protocol.

Week	Intervention
I	Instructions about the correct technique of performing the “foot shortening” and MLA elevation by drawing the first metatarsal head towards the calcaneus, without curling the toes. Sitting position. The patient can raise the toes from the floor while leaving the heads of the metatarsals in contact with the floor and then slowly lowering the toes to the floor while maintaining the height of the medial longitudinal arch.
II	Performing “foot shortening” and MLA elevation by drawing the first head of the metatarsal bone towards the calcaneus, without curling the toes. Sitting position. The patient cannot lift his fingers off the floor. The use of feedback stabilization on the knee joints.
III	Performing the “foot shortening” and MLA elevation by drawing the first metatarsal head towards the calcaneus, without curling the toes. Standing position. The patient can raise the toes from the floor while leaving the heads of the metatarsals in contact with the floor and then slowly lowering the toes to the floor while maintaining the height of the medial longitudinal arch.
IV	Performing “foot shortening” and MLA elevation by drawing the first head of the metatarsal bone towards the calcaneus, without curling the toes. Standing position. The patient cannot lift his fingers off the floor.
V	Performing “foot shortening” and MLA elevation by drawing the first head of the metatarsal bone towards the calcaneus, without curling the toes. The lunge position. The patient cannot lift his fingers off the floor. They exercise both feet simultaneously. After 20 repetitions, the front limb is changed.
VI	Instruction and explanation of the use of “foot shortening” and rise the MLA during walking in the appropriate sub-phases of the stance phase.

MLA—Medial Longitudinal Arch.

**Table 2:** Percentage of unsupervised SFE rehabilitation program at home by a given group each week.

Group	I	II	III	IV	V	VI
EBW	81.90%	87.62%	87.62%	80.00%	81.90%	87.62%
NBW	85.71%	87.62%	86.67%	88.57%	87.62%	92.38%

EBW—Excessive Body Weight Group, NBW—Normal Body Weight Group.

**Table 3:** The demographic data of children.

Demographic Data	EBW	NBW	CG
BMI	24.9 ± 4.0	16.9 ± 1.6 *	17.8 ± 2.4 **
Age	10.1 ± 1.8	9.4 ± 1.9	9.3 ± 1.9

EBW—Excessive Body Weight Group, NBW—Normal Body Weight Group, CG—Control Group. Significantly different to EBW group: * NBW/EBW *p* = 0.000, ** CG/EBW *p* = 0.000.

**Table 4:** A stability index in the range of 8–12 platform mobility levels.

Stability Index	EBW		*p*-Level	NBW		*p*-Level	CG
	1	2		1	2		
OSI	1.89 ± 0.74	1.24 ± 0.66	0.997	1.23 ± 1.15	0.98 ± 0.53	0.519	0.81 ± 0.36
AP	1.40 ± 0.61	0.92 ± 0.52	0.996	0.92 ± 1.12	0.76 ± 0.52	0.857	0.57 ± 0.33
ML	1.01 ± 0.34	0.63 ± 0.33	1.000	0.64 ± 0.41	0.47 ± 0.19	0.150	0.45 ± 0.23

Overall Stability Index (OSI), Anterior-Posterior Stability Index (APSI), and Medio-Lateral Stability Index (MLSI), with eyes open. EBW—Excessive Body Weight Group, NBW—Normal Body Weight Group, CG—Control Group. The NBW1, and EBW1 groups before rehabilitation and the NBW2 and EBW2 groups after rehabilitation.

**Table 5:** A stability index in the range 5 platform mobility level.

Stability Index	EBW		*p*-Level	NBW		*p*-Level	CG
	1	2		1	2		
OSI	1.65 ± 2.16	0.90 ± 0.45	0.236	0.89 ± 0.72	0.73 ± 0.50	0.996	0.66 ± 0.20
AP	1.23 ± 1.55	0.64 ± 0.32	0.206	0.70 ± 0.74	0.55 ± 0.47	0.992	0.50 ± 0.17
ML	0.88 ± 1.35	0.48 ± 0.26	0.382	0.40 ± 0.22	0.37 ± 0.25	1.000	0.33 ± 0.15

Overall Stability Index (OSI), Anterior-Posterior Stability Index (APSI), and Medio-Lateral Stability Index (MLSI), with eyes open. EBW—Excessive Body Weight Group, NBW—Normal Body Weight Group, CG—Control Group.

**Table 6:** Pearson linear correlation between BMI and OSI, in tests 8–12 and test 5.

Pearson Linear Correlation	Test 8–12		Test 5	
	r	*p*-Level	r	*p*-Level
BMI—OSI	0.43	0.0001	0.44	0.0001
BMI—OSI eyes open	0.42	0.001	0.35	0.006
BMI—OSI eyes closed	0.55	0.0001	0.60	0.0001

## Data Availability

The data presented in this study are available upon request from the corresponding author.
